# Brain: biomedical knowledge manipulation

**DOI:** 10.1093/bioinformatics/btt109

**Published:** 2013-03-16

**Authors:** Samuel Croset, John P. Overington, Dietrich Rebholz-Schuhmann

**Affiliations:** EMBL European Bioinformatics Institute, Wellcome Trust Genome Campus, Hinxton, Cambridge, CB10 1SD, UK

## Abstract

**Summary:** Brain is a Java software library facilitating the manipulation and creation of ontologies and knowledge bases represented with the Web Ontology Language (OWL).

**Availability and implementation:** The Java source code and the library are freely available at https://github.com/loopasam/Brain and on the Maven Central repository (GroupId: uk.ac.ebi.brain). The documentation is available at https://github.com/loopasam/Brain/wiki.

**Contact:** croset@ebi.ac.uk

**Supplementary information:** Supplementary data are available at *Bioinformatics* online.

## 1 MOTIVATION

Knowledge bases, a concept from computer science (Krötzsch *et al.*, 2012 for an introduction), could be a solution to improve the interoperability and the value of the large amount of biomedical information available online. At the time of writing, a few options are available to handle such knowledge bases: complex libraries as the Web Ontology Language (OWL)-API (Horridge and Bechhofer, 2011) or didactic graphical user interfaces, such as Protege (http://protege.stanford.edu/) or TopBraid Composer (http://www.topbraidcomposer.com). An intermediary framework, OWLTools (http://code.google.com/p/owltools/), provides some methods to query biomedical ontologies, but the interaction with the library is mostly done via command-lines, which limits the scale of projects that can be built with it. Brain—the Java software library presented in this manuscript addresses these issues and provides a comprehensive and simplified interface, dedicated to the programmatic creation and query of biomedical knowledge bases. The library aims at bridging the gap between graphical user interfaces and the OWL-API and is particularly useful to develop web applications. Brain has a particular focus on the EL profile of OWL, as it covers the majority of biomedical use-cases and unlocks good reasoning performance and scalability.

## 2 SCALABLE KNOWLEDGE BASES

**Fig. 1.**
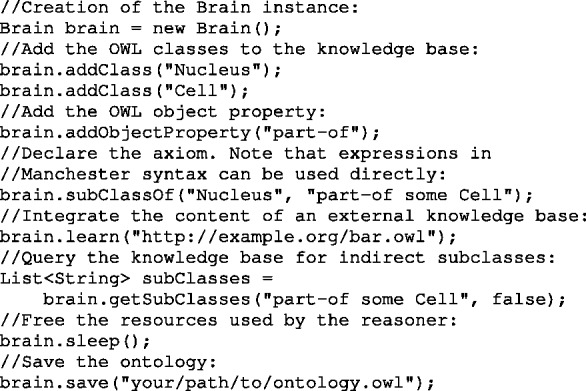
Implementation example in Java of an axiom using Brain; the axiom expressed in natural language: *A nucleus is part of some cells*. Same axiom described in OWL using the Manchester syntax: *Nucleus subClassOf part-of some Cell*

The OWL derives from description logic and has been designed to capture the knowledge of a domain of interest in the form of a structured vocabulary (http://www.w3.org/TR/owl2-overview/). This feature makes it particularly interesting from the perspective of the life sciences, as a number of ontologies and classification schemes have been developed from the origin of the discipline and can now be converted into an OWL representation. Brain focuses on a particular profile of OWL, called EL, which consists of a subset of the constructs available in the original language (Motik *et al.*, 2009). This profile is designed to be *tractable*, meaning that the axioms available have a polynomial complexity and are, therefore, easier to compute than the full version of OWL. Brain primarily supports the OWL 2 EL profile for its computational properties and suitability for real-life biomedical applications, where millions of axioms could be potentially extracted from complex repositories, such as ChEMBL (Gaulton *et al.*, 2012). Moreover, the EL profile is expressive enough to cover a good portion of biomedical knowledge: most of Open Biomedical Ontologies (OBO), such as the Gene Ontology (GO—Ashburner *et al.*, 2000) or the Chemical Entities of Biological Interest (ChEBI—De Matos *et al.*, 2010), are already included in this profile, opening doors to large-scale meaningful data integration. Brain builds on the top of Elk, a fast reasoner dedicated to EL ontologies (Kazakov *et al.*, 2011). Elk shows good performances at handling large datasets and offers the possibility of running some reasoning tasks in parallel; therefore, clusters or multicore architecture can scale the speed of reasoning as more data are added to the knowledge base. Brain wraps and simplifies the interaction with Elk while still leaving the possibility to fine-tune the configuration for advanced users. Figure 1 shows an example of query using the Elk reasoner.

## 3 LIBRARY FEATURES

Brain is implemented as a facade leveraging the access to the OWL-API and providing a series of convenience methods for common use-cases encountered in the biomedical domain. To simplify the interaction with the OWL-API, Brain follows a series of features described later in the text. The full list of currently supported constructs and methods is available in the Supplementary Material and in the online documentation.

### 3.1 Unique knowledge base

An instance of a Brain object holds a reference to only one knowledge base. It is yet possible to import some external ontologies, either stored locally or via a network, but Brain will always merge the added information to the existing knowledge base.

### 3.2 Unique short form names

The names (short forms) of OWL entities handled by a Brain object have to be unique. It is for instance not possible to add an OWL class, such as http://www.example.org/Cell to the ontology if an OWL entity with the short form ‘Cell’ already exists. Despite being in contradiction with some Semantic Web principles, this design prevents ambiguous queries and hides as much as possible the cumbersome interaction with prefixes and Internationalized Resource Identifiers (IRI).

### 3.3 Typeless interaction

The interaction with the library relies on the user-friendly Manchester syntax entered as string (Horridge *et al.*, 2006). This permits moving away from the creation of Java objects and is particularly suitable in a web server set-up where requests are likely to be some typeless characters. Using strings as input also speeds the production and flexibility of the code written, when moving from a relational database to an OWL representation, for example. Figure 1 provides an example of axiom implementation using the Manchester syntax.

### 3.4 Error-handling

Because the interaction with Brain is built around strings rather than Java objects, special care has to be put on exceptions handling to safely maintain the correct execution of the program. Brain throws different types of error tailored to the operation performed by the user. This feature is mandatory while developing large applications and helps to maintain the consistency of the underlying knowledge base.

### 3.5 Knowledge integration

An interesting feature brought by the Semantic Web and OWL is the possibility to merge information based on the IRIs of the entities described. The library supports the loading and integration of external knowledge bases, as well as references to external entities. Data from different sources can, therefore, be easily connected and reason over by Brain. The integration of an external knowledge base is shown on Figure 1.

### 3.6 Querying

Brain is oriented towards efficient querying of OWL 2 EL knowledge bases. This characteristic makes it suitable as a query engine on a web server for answering live queries from users. Powerful questions can be formulated using the Manchester syntax, abstracting away complex interaction with the Java objects provided by the OWL-API (illustrated in Fig. 1). An example of question answering over the GO using Brain is compared with a traditional SQL query in the Supplementary Material.

## 4 CONCLUSION

Brain is an open source Java library designed to build and query biomedical knowledge bases or OWL ontologies. The library is centered on the EL profile and designed to be suitable and scalable for biomedical knowledge representation. The convenience methods provided by Brain should simplify the development of biomedical knowledge bases and allow developers to increase their productivity while effectively dealing with data integration challenges.

*Funding*: EMBL member states. S.C. is a member of Darwin College, University of Cambridge.

*Conflict of Interest*: none declared.

## Supplementary Material

Supplementary Data
