# Supplementary material for: Brain: biomedical knowledge manipulation
Source: Bioinformatics. 2013 Mar 16;29(9):1238–9. doi: 10.1093/bioinformatics/btt109 (PMC3634181; doi:10.1093/bioinformatics/btt109)
Supplement: Supplementary Data [file supp_29_9_1238__index.html]

Brain: Biomedical Knowledge Manipulation — Brain: biomedical knowledge manipulation — Brain: biomedical knowledge manipulation — Supplementary Data 

# Brain: biomedical knowledge manipulation

## Supplementary Data

files

**Files in this Data Supplement:**

- Supplementary Data - pdf file
