# Supplementary material for: Brain: biomedical knowledge manipulation
Source: Bioinformatics. 2013 Mar 16;29(9):1238–9. doi: 10.1093/bioinformatics/btt109 (PMC3634181; doi:10.1093/bioinformatics/btt109)
Supplement: Supplementary Data [file supp_btt109_supplementary-croset.pdf]

Supplementary Material

Table 1: Example of some common OWL 2EL constructs written using the Manchester syntax alonside an example of implementation using Brain.

Name	Description Logic	OWL (Manchester Syntax)	Brain implementation
**Concepts**			
atomic concept	A	Class: A	brain.addClass("A");
intersection	$C \sqcap D$	C and D	brain.equivalentClasses("A","C and D");
top concept	$\top$	owl:Thing	brain.getOWLClass("Thing");
bottom concept	$\bot$	owl:Nothing	brain.getUnsatisfiableClasses();
union	$C \sqcup D$	C or D	Not supported (Not in EL profile)
complement	$\neg C$	not C	Not supported (Not in EL profile)
existential restriction	$\exists R.C$	P some C	brain.subClassOf("A", "P some C");
universal restriction	$\forall R.C$	P only C	Not supported (Not in EL profile)
**Roles**			
atomic role	R	ObjectProperty: P	brain.addObjectProperty("P");
**Individuals**			
individual name	a	Individual: a	Not supported yet
**Axioms**			
TBox (terminological axioms)			
concept inclusion	$C \sqsubseteq D$	C SubClassOf: D	brain.subClassOf("C", "D");
concept equivalence	$C \equiv D$	C EquivalentTo: D	brain.equivalentClasses("C", "D");
concept disjointness	$C \sqcap D \sqsubseteq \bot$	C DisjointWith: D	brain.disjointClasses("C", "D");
RBox (relational axioms)			
role inclusion	$R \sqsubseteq S$	R SubPropertyOf: S	brain.subPropertyOf("R", "S");
role equivalence	$R \equiv S$	R EquivalentTo: S	brain.equivalentProperties("R", "S");
complex role inclusion	$R1 \circ R2 \sqsubseteq S$	S SubPropertyChain: R1 o R2	brain.chain("R1 o R2", "S");
role transitivity	$R \circ R \sqsubseteq R$	Characteristics: Transitive	brain.transitive("R");
ABox (assertional axioms)			
concept assertion	C(a)	a Types: C	Not supported yet
role assertion	R(a, b)	a Facts: R b	Not supported yet
individual equality	$a = b$	a SameAs: b	Not supported yet
individual inequality	$a \neq b$	a DifferentFrom: b	Not supported yet

Table 2: The same query involving implicit knowledge retrieval is formulated using SQL and OWL over the Gene Ontology (GO). The original data comes from the website of the GO: http://www.geneontology.org/GO.downloads.database.shtml

	**SQL**	**OWL (via Brain)**
Source	go_daily-termdb-tables/	go_daily-termdb.owl
Access	http://www.berkeleybop.org/goose/	brain.learn("go_daily-termdb.owl");
Query:<br>Explicit and implicit<br>regulators of 'blood coagulation'	SELECT DISTINCT * FROM term<br>INNER JOIN graph_path AS g<br>ON (term.id=g.term1_id<br>AND g.relationship_type_id=21)<br>INNER JOIN term AS r<br>ON (r.id=g.term2_id)<br>WHERE term.name='blood coagulation'<br>AND distance <> 0 ;	brain.getSubClasses(<br>"RO_0002211 some GO_0007596",<br>false);
Method of retrieval	Iteration over<br>additional tables storing all<br>the possible links (closure graph)	Automated reasoning<br>over the knowledge base<br>using an abstract OWL expression
